# Meaningful Criteria to Persons Living With Cystic Fibrosis and Their Healthcare Providers in Helping Determine Adjustments to Routine Clinical Follow‐Up

**DOI:** 10.1002/ppul.71729

**Published:** 2026-07-10

**Authors:** William R. Hunt, Christian Merlo, Ashley Keller, Karen Lowe, Kristin Riekert, James D. Finklea

**Affiliations:** ^1^ Emory University Atlanta Georgia USA; ^2^ Johns Hopkins University Baltimore Maryland USA; ^3^ Department of Internal Medicine University of Texas Southwestern Dallas Texas USA

**Keywords:** Care Model, Cystic Fibrosis, Highly effective modulator therapy

## Abstract

**Introduction:**

Persons living with cystic fibrosis (PwCF) have experienced fewer exacerbations and symptom burden over the last decade, largely thanks to widespread uptake of highly effective modulator therapy (HEMT). With these advancements, there have been interest in the community regarding adjustments to the care model. However, there is a paucity of data with which to guide discussions for clinical follow‐up.

**Objective:**

The goal of the survey is to better understand the components of clinical care that are perceived as important when PwCF and their healthcare providers are considering the timing of routine clinical evaluations.

**Methods:**

The survey asked predominantly United States PwCF and healthcare providers via CF Community listservs which factors should be considered when determining the interval between routine clinic visits. These included pulmonary function, respiratory symptoms, use of HEMT, pulmonary exacerbations (PExs), and co‐morbidities.

**Results:**

The survey was completed by 152 PwCF and 177 medical providers. Pediatric providers and PwCF were comfortable with higher baseline lung function in comparison to adult providers when considering visit extensions. PwCF and pediatric providers felt more frequent visits were required in comparison to adult providers. PwCF were more tolerant of prior PExs requiring IV antibiotics. There were no differences between groups with respect to the maximum number of PExs requiring oral antibiotics in the previous year to safely extend the interval between visits.

**Conclusion:**

The survey identified and quantified factors that PwCF, adult providers, and pediatric providers felt were important when considering adjusting the interval between clinic visits.

**Abbreviations**
CF: Cystic fibrosis
CFF: CF Foundation (CFF)
CFFPRD: Cystic Fibrosis Foundation Patient Registry Database
CFRD: CF related diabetes
FEV_1_pp: Forced expiratory volume in 1 s percent predicted
HEMT: Highly‐effective modulator therapy
NTM: Nontuberculous mycobacterium
PEx: Pulmonary exacerbation
PwCF: Persons with CF
TXP: Transplantation

## 1 Introduction

The advent of highly effective modulator therapy (HEMT) led to a paradigm shift in cystic fibrosis (CF) therapeutics and, consequently, a major modification in the prognosis of persons living with CF (PwCF) [1]. For PwCF who have responsive CF variants, HEMT has been shown to reduce pulmonary exacerbations (PExs), improve lung function, and significantly reduce respiratory symptom scores [2, 3, 4]. With the majority of PwCF benefiting from HEMT use, many are choosing to reduce the number of CF‐specific treatments that make up their daily therapeutic routine [5, 6].

Shortly after the widespread availability of HEMT in the United States (late winter/early spring of 2020), the SARS CoV‐2 (COVID‐19) pandemic spread across the globe. The pandemic necessitated a substantial change in the routine CF care model nearly overnight [7]. CF care centers quickly adopted telehealth visits as an alternative model of care [8, 9, 10]. The combination of the global pandemic and the use of HEMT led PwCF to reduce the number of clinic visits.

Prior to widespread use of HEMT, the United States CF Foundation (CFF) recommended quarterly visits for PwCF to ensure pulmonary function stability, adherence to the chronic respiratory therapy, and to educate PwCF as one ages [11]. Interestingly, CFF Patient Registry Data (CFFPRD) indicate that despite a reduction in quarterly visits since 2020, the number of PExs decreased while lung function actually improved as a consequence of HEMT use [6]. As such, thoughtful consideration has turned towards evaluating the appropriateness of the traditional CF care model. In 2024, the CFF published a position paper from a multi‐disciplinary expert panel that explored potential adaptations to the traditional care model. The position paper recommends PwCF attend clinic at least every 4–6 months if one's overall health is stable, which would be a reduction from 4 clinic visits per year to 2–3 per year. Some criteria that would preclude extending the interval between visits are a new CF diagnosis, such as CF related diabetes (CFRD), or life transitions (having your first child) [12]. However, clinical parameters deemed important during routine care adjustment considerations may be different between PwCF and healthcare providers. The goal of this project was to survey PwCF and a wide sample of CF providers to determine which clinical parameters were important to their discussions regarding adjustments to routine care follow‐up.

## 2 Methods

### 2.1 Survey Development

This study was a sampling survey to explore health‐related parameters PwCF and CF healthcare providers deemed important when discussing altering routine clinic follow‐up. Two surveys (one for PwCF and one specific for CF healthcare providers) were developed via a multidisciplinary discussion including three CF physicians (two adult internists, one internist and pediatrician), a psychologist with extensive experience in CF research and survey development, and a patient representative living with CF. The PwCF survey was written at an 8–9th grade level, while the medical provider survey was at a college level according to the Flesch‐Kincaid calculator [13]. Survey components measured the degree of importance respondents assigned to clinical parameters such as: presence of specific CF diagnoses, CFTR modulator use, lung function baseline, symptom burden, adherence to chronic therapies, and history of PExs when considering altering routine follow‐up. These criteria aligned with factors in determining if one had stable health based on the recent CFF position paper on redefining the CF care model [12]. Response options were primarily Likert scale (not at all = 1, a little = 2, somewhat = 3, greatly = 4, and completely = 5), open‐ended responses and a scale to determine the severity of PwCF cough (no pulmonary symptoms (e.g., never coughs or coughs once every few days), minimal pulmonary symptoms (e.g., coughs a few times during the day), mild pulmonary symptoms (e.g., coughs only in the morning after waking up), moderate pulmonary symptoms (e.g., coughs several times a day), and persistent pulmonary symptoms (e.g., coughs at least once to multiple times every hour). Participants were also queried to share their lower tolerance level of lung function baseline (forced expiratory volume in 1 s percent predicted (FEV_1_pp), upper tolerance level of specific symptom burden, and the number of recent PExs one would still feel safe with considering altering routine follow‐up. Survey questions for the two cohorts (PwCF and CF providers) mirrored one another, with the exception that the CF provider survey had one additional question exploring the perceived severity of different CF diagnoses and whether the presence of those diagnoses would affect their comfort with altering routine follow‐up (see “Supplementary Materials”).

Study data were captured through REDCap surveys (Research Electronic Data Capture, hosted at University of Texas Southwestern (UTSW)) and were distributed to individuals in the CF community via CFF Community Voice, CF Physician Listserv, and CF Advanced Practice Provider Listserv. Survey collection occurred between January 15th to April 30th, 2024. Surveys were also distributed to PwCF via a QR CODE at three large academic adult CF programs across the southeastern and mid‐Atlantic United States (Emory University, Johns Hopkins University, and the UTSW Adult CF Clinics).

### 2.2 Statistical Analysis

All statistical analyses were performed in SPSS Version 28.0.1.0. Data is presented as mean ± standard deviation or number (percentage). Ordinal, Likert responses between cohorts were analyzed by the Kruskal–Wallis test based on ranks with Bonferroni correction for multiple comparisons. Two‐sample *t* tests were used to compare differences of continuous variables between the survey cohorts (adult PwCF compared to adult providers and pediatric provider to adult providers). Finally, differences in categorical variables were analyzed for the CF provider cohorts utilizing a 2‐sided asymptotic Pearson Chi‐Square Test. Alpha was set at 0.05.

### 2.3 Ethical Approval

The UTSW Institiutonal Review Board approved all study procedures (study identification number; STU‐2023‐0257). The study was determined to be exempt because the survey did not collect identifiable data. Thus, informed consent was not required. Additionally, the study was deemed to be minimal risk because the survey did not collect identifiable data that would allow human subjects to be ascertained.

## 3 Results

The survey was completed by 152 PwCF and 177 healthcare providers. In regard to healthcare providers, 92 practiced pediatrics and 85 practiced adult medicine. Of PwCF, 83% of respondents endorsed being on HEMT, with 100% attending adult clinics.

PwCF placed less importance on previous PEx (*p* < 0.05) than adult providers when contemplating their perceived comfort in altering routine clinic follow‐up. All other factors, including lung function stability, active respiratory symptoms, CF co‐morbidities, use of HEMT, and adherence with therapies, were not statistically different when comparing PwCF to adult providers and pediatric to adult providers (Table 1). Participants were also queried as to their tolerance of baseline lung function (FEV_1_pp), specific symptom burden and number of recent PExs in their considerations of altering routine follow‐up. Adult providers were comfortable with a lower baseline FEV_1_pp on average of 54.4 ± 14.3% in comparison to PwCF (FEV_1_pp 67.9 ± 24.5%; *p* < 0.001) and to pediatric providers (77.96 ± 13.2%; *p* < 0.001) when considering extending time to the next clinic visit. Additionally, adult providers favored a lower minimal number of annual clinic visits (2.17 ± 0.71 per year) in comparison to PwCF (2.79 ± 1.23; *p* < 0.001) and pediatric providers (2.75 ± 0.75; *p* < 0.001).

**Table 1:** Importance assigned to specific factors by survey participants when considering alterations to routine follow‐up.

		Not at all	A little	Somewhat	Greatly	Completely	*p* value*
Lung Function	PwCFα (*n* = 152)	4 (2.6%)	12 (7.8%)	22 (14.5%)	53 (34.9%)	61 (40.1%)	α NS; β NS
	Pediatric Providersβ (*n* = 92)	0 (0%)	2 (2.2%)	6 (6.5%)	45 (48.9%)	39 (42.4%)	
	Adult Providers (*n* = 85)	0 (0%)	1 (1.2%)	9 (10.6%)	48 (56.5%)	27 (31.8%)	
Pulmonary Exacerbations	PwCFα (*n* = 149)	14 (9.4%)	12 (8.1%)	18 (12.1%)	53 (35.6%)	52 (34.9%)	α < 0.05; β NS
	Pediatric Providersβ (*n* = 92)	2 (2.2%)	0 (0%)	8 (8.7%)	44 (47.8%)	38 (41.3%)	
	Adult Providers (*n* = 85)	0 (0%)	0 (0%)	3.5 (3%)	50 (58.8%)	32 (37.6%)	
Resp Symptoms	PwCFα (*n* = 151)	7 (4.6%)	16 (10.6%)	32 (21.2%)	56 (37.1%)	40 (26.5%)	α NS; β NS
	Pediatric Providersβ (*n* = 91)	1 (1.1%)	0 (0%)	13 (14.3%)	47 (51.6%)	30 (33%)	
	Adult Providers (*n* = 84)	0 (0%)	0 (0%)	18 (21.4%)	46 (54.8%)	20 (23.8%)	
Co‐Morbidities	PwCFα (*n* = 147)	9 (6.1%)	19 (12.9%)	30 (20.4%)	52 (35.4%)	37 (25.2%)	α NS; β NS
	Pediatric Providersβ (*n*‐92)	1 (1.1%)	0 (0%)	18 (19.6%)	47 (51.1%)	26 (28.3%)	
	Adult Providers (*n* = 85)	0 (0%)	2 (2.4%)	17 (20%)	49 (57.6%)	17 (20%)	
HEMT	PwCFα (*n* = 147)	23 (15.6%)	14 (9.5%)	24 (16.3%)	36 (24.5%)	50 (34%)	α NS; β NS
	Pediatric Providersβ (*n* = 92)	0 (0%)	2 (2.2%)	18 (19.6%)	38 (41.3%)	34 (37%)	
	Adult Providers (*n* = 85)	0 (0%)	3 (3.5%)	24 (28.2%)	36 (42.4%)	22 (25.9%)	
Adherence	PwCFα (*n* = 149)	22 (14.8%)	11 (7.4%)	28 (18.8%)	42 (28.2%)	46 (30.9%)	α NS; β NS
	Pediatric Providersβ (*n* = 91)	0 (0%)	3 (3.3%)	22 (24.2%)	44 (48.4%)	22 (24.2%)	
	Adult Providers (*n* = 85)	0 (0%)	7 (8.2%)	24 (28.2%)	37 (43.5%)	17 (20%)	

*Note: Questions*: For an established PwCF who, in your judgment, is currently at their “baseline” and clinically stable, to what extent do/would you consider the following factors when deciding if it is safe to extend the clinic visit interval to more than three months?

Likert scale is presented at number (%). The Likert scale numbers are (not at all = 1, a little = 2, somewhat = 3, greatly = 4, and completely = 5).

* *p* values represent independent samples Kruskal–Wallis test, significance values have been adjusted by the Bonferroni correction for multiple comparisons.

^α^ Adult Providers to PwCF.

^β^ Adult Providers to Pediatric Providers.

Regarding PExs, there were no differences between groups when compared to adult providers in their maximal tolerance of PEx requiring oral antibiotics within the preceding 12 months (Table 2). However, PwCF were comfortable having more courses of IV antibiotics (1.29 ± 1.29 within the preceding 12 months) in comparison to adult providers (0.78 ± 0.66; *p* < 0.05). There were no differences between adult and pediatric providers' tolerance of IV antibiotic use within the preceding 12 months when considering possibilities of adjusting routine follow‐up (Table 2).

**Table 2:** PwCF and medical providers opinions on the criteria that must be met to safely extend the interval between visits.

		PwCFα	Pediatric providersβ	Adult providers	*p* value
Lowest FEV1pp (%)		67.9 ± 24.5 (*n* = 121)	77.96 ± 13.2 (*n* = 81)	54.4 ± 14.3 (*n* = 79)	α < 0.001 β < 0.001*
Minimum number of annual visits	Average 1	2.79 ± 1.23 13 (9%)	2.75 ± 0.75 0 (0%)	2.17 ± 0.71 10 (12%)	α < 0.001; β < 0.001*
	2	48 (33.1%)	38 (43.2%)	51 (61.4%)	
	3	27 (18.6%)	34 (38.6%)	16 (19.3%)	
	4 or more	57 (39.3%)	12 (18.2%)	6 (7.2%)	
Maximum number of course of IV antibiotics within the Last 12 months	Average 0	1.29 ± 1.29 34 (27.6%)	0.64 ± 0.57 37 (40.7%)	0.78 ± 0.66 29 (34.9%)	α < 0.05; β NS*
	1	49 (39.8%)	50 (54.9%)	42 (51.8%)	
	2	23 (18.7%)	4 (4.4%)	11 (13.3%)	
	3 or more	17 (13.9%)	0 (0%)	0 (0%)	
Maximum number of course of oral antibiotics within the last 12 months	Average 0	1.89 ± 1.43 18 (13.5%)	1.84 ± 0.87 6 (6.6%)	1.95 ± 0.83 5 (6.2%)	α NS; β NS*
	1	36 (27.1%)	22 (24.2%)	13 (16%)	
	2	45 (33.8%)	47 (51.6%)	50 (61.7%)	
	3 or more	34 (25.6%)	16 (17.6%)	13 (16.1%)	

*Note:* Data presented as number (%).* Independent samples Kruskal–Wallis test, significance values have been adjusted by the Bonferroni correction for multiple comparisons.

^α^ Comparison of Adult Providers to PwCF.

^β^ Comparison of Adult Providers to Pediatric Providers.

PwCF and adult providers agreed with the severity of cough that one could extend the interval between visits (*p* = 0.60). However, pediatric providers favored “no” to “minimal” levels of coughing prior to considering extending interval between visit in comparison to adult providers who were tolerant of “mild” cough levels (*p* < 0.001) (Table 3).

**Table 3:** Maximal daily pulmonary symptoms that survey participants would consider safe to consider adjusting routine follow‐up.

	PwCFα (*n* = 145)	Pediatric providersβ (*n* = 92)	Adult providers (*n* = 85)	*p* value
No pulmonary symptoms	21 (14.5%)	90 (31.5%)	5 (5.9%)	α NS; β < 0.001
Minimal pulmonary symptoms	45 (31%)	34 (37%)	23 (27.1%)	
Mild pulmonary symptoms	52 (35.9%)	27 (29.3%)	47 (55.3%)	
Moderate pulmonary symptoms	25 (17.2%)	2 (2.2%)	9 (10.6%)	
Persistent pulmonary Symptoms	2 (1.4%)	0 (0%)	1 (1.2%)	

*Note:* Likert scale data is presented as number (%). The severity of cough was based on the following:

No pulmonary symptoms (e.g., never coughs or coughs once every few days).

Minimal pulmonary symptoms (e.g., coughs a few times during the day).

Mild pulmonary symptoms (e.g., coughs only in the morning after waking up).

Moderate pulmonary symptoms (e.g., coughs several times a day).

Persistent pulmonary symptoms (e.g., coughs at least once to multiple times every hour).

Independent samples Kruskal–Wallis Test, pairwise comparisons, asymptotic significances (2‐sided), adjusted by the Bonferroni correction for multiple comparisons.

^α^ PwCF‐Adult – NS.

^β^ Pediatric‐Adult – *p* < 0.001.

Providers were also asked to evaluate how important the presence or absence of specific diagnoses are when considering altering routine care follow‐up (Table 4). At least 70% of medical providers considered chronic hypercapnic respiratory failure (91.5%), chronic hypoxic respiratory failure (90.4%), massive hemoptysis in the last 12 months (90.4%), active treatment for allergic bronchopulmonary *Aspergillosis* (88.7%), body mass index ≤ 18.5 kg/m^2^ (88.1%), uncontrolled asthma (84.2%), pneumothorax in the last 12 months (81.9.%), pregnancy (77.4%), culture positive for *Nontuberculous mycobacterium* (NTM) (76.3%), CFRD with an A1c ≥ 7.5% (73.4%), history of solid organ or hematological transplantation (TXP) (72.9%), and moderate/severe depression symptoms (70.0%) to be important factors to consider. Pediatric practitioners, in comparison to adult providers, viewed *Burkholderia* colonization or infection (66.3% vs 49.4%; *p* < 0.05), CFRD (79.3% vs 67.1%; *p* < 0.05), and NTM colonization or infection (85.8% vs 65.8%; *p* < 0.01) with higher importance when considering the likelihood of extending the interval between clinic visits. Adult providers, in comparison to pediatric providers, felt that pregnancy (88.2% vs 67.4%; *p* < 0.001) would weigh higher in their contemplation for extending routine visits.

**Table 4:** Provider report of co‐morbidities that would decrease the likelihood of extending a CF clinic visit interval.

	All providers (*N* = 177)	Pediatric providers (*N* = 92)	Adult providers (*N* = 85)	*P* value
Active treatment for Allergic Bronchopulmonary Aspergillosis	157 (88.7%)	83 (90.2%)	74 (87.1%)	NS
Moderate/severe anxiety symptoms	108 (61.0%)	56 (60.9%)	52 (61.2%)	NS
Uncontrolled asthma	149 (84.2%)	74 (80.0%)	75 (88.2%)	NS
Body mass index less than 18.5 kg/m^2^	156 (88.1%)	83 (90.2%)	73 (85.9%)	NS
Body mass index greater than 30 kg/m^2^	27 (15.3%)	16 (17.4%)	11 (13.0%)	NS
*Burkholderia* colonization or infection	103 (58.2%)	61 (66.3%)	42 (49.4%)	< 0.05
Chronic fungal (*Trichosporon* or *Scedosporium*) colonization or infection	54 (30.5%)	29 (31.5%)	25 (29.4%)	NS
Chronic hypercapnic respiratory failure	162 (91.5%)	85 (92.4%)	77 (90.6%)	NS
Chronic hypoxic respiratory failure	160 (90.4%)	86 (93.5%)	74 (87.1%)	NS
Chronic sinusitis	14 (7.9%)	10 (10.9%)	4 (5.0%)	NS
Constipation	16 (9.0%)	7 (8.0%)	9 (11.0%)	NS
Cystic Fibrosis‐related diabetes with an A1c above 7.5%	130 (73.4%)	73 (79.3%)	57 (67.1%)	< 0.05
Moderate/severe depression symptoms	124 (70.0%)	67 (72.8%)	57 (67.1%)	NS
Distal intestinal obstruction syndrome in the last 12 months	92 (52.0%)	51 (55.4%)	41 (48.2%)	NS
History of solid organ or hematological transplantation	129 (72.9%)	76 (82.6%)	53 (62.4%)	< 0.01
Massive Hemoptysis in the last 12 months	160 (90.4%)	86 (93.5%)	74 (87.1%)	NS
Nontuberculous mycobacterium colonization or infection	135 (76.3%)	79 (85.8%)	56 (65.8%)	< 0.01
Pneumothorax in the last 12 months	145 (81.9%)	78 (84.8%)	67 (78.8%)	NS
Pregnancy	137 (77.4%)	62 (67.4%)	75 (88.2%)	< 0.001

*Note:* Data are presented as *n* (%); *p*‐values represent Chi‐square comparisons.

## 4 Discussion

The survey results' major findings are that both PwCF and healthcare providers had a perceived comfort level with extending the interval between visits in specific circumstances if certain criteria are met. All participants felt PwCF could reduce the number of clinic visits with adult providers, respondents being more comfortable with having fewer visits than PwCF and pediatric providers.

One of the potential key factors in determining if one can extend the interval between visits is lung function. While there are many ways of evaluating lung function, FEV_1_pp, as determined by spirometry, has been a key clinical and research metric used in PwCF for decades. In this survey, both PwCF and healthcare providers put a similar high value on including lung function when considering changes to the interval between visits (Table 1). Survey respondents selected similar levels for minimum required FEV_1_pp when considering extending the clinic interval, with an average of 67.03 ± 21.08%. However, there were differences between the healthcare provider cohorts, with pediatric providers favoring a higher FEV_1_pp while adult providers were comfortable with a lower FEV_1_pp. The higher value for pediatric providers is not surprising and likely reflects experience with the expectation for younger patients to have higher lung function at that time point in their lives. It is important to note that while the age of respondents was not recorded in this survey, all PwCF respondents reported attending adult CF clinics. Therefore, the majority, if not all, PwCF respondents were likely over 18. Thus, a broader sampling of youth with CF and their caregivers may find significant differences in the value they would place on lung function.

There are a number of clinical trials exploring reductions to CF therapeutic regimens that have included lung function baseline as either a safety endpoint or inclusion criteria. The SIMPLIFY study explored treatment de‐escalation in individuals stable on HEMT. Participants included in the study were randomized to discontinue either inhaled hypertonic saline or dornase alfa for 6 weeks. In this study, researchers selected a lower limit for FEV_1_pp of 70% for participants 12 years old and up and 60% for adults who were 18 years and older [14]. The CF – STORM study was a pragmatically designed study to also explore the feasibility of therapy de‐escalation, similar to the SIMPLIFY study, but with a longer follow‐up of 12 months. Participants again were randomized to discontinue either hypertonic saline or dornase alfa in PwCF 6 years and older with an FEV_1_pp inclusion criteria of ≥ 40% [15]. Guenther et al. conducted a single‐center retrospective study where providers and PwCF used a de‐escalation algorithm to reduce respiratory therapies burden. The median baseline FEV_1_pp was 75 ± 26% after 1 month of ETI [16]. The Home‐Reported Outcomes with CFTR Modulator Therapy (HERO2) is an ongoing observational open‐label trial evaluating self‐discontinuation rates of respiratory therapies in PwCF. While only an observational, non‐intervention study, baseline enrollment characteristics of the participants for the study noted that there was no minimal lung function for study inclusion. Of those who enrolled in HERO2, 24.7% participants had an FEV_1_pp less than 70%, and 7.0% were less than 40% [17]. Finally, the REFORM study is actively enrolling adults with CF in Australia who meet prespecified criteria for randomization to see if routine follow‐up visits every 6 months is non‐inferior to visits every 3 months. The FEV_1_pp must be ≥ 40% to enroll in this study [18]. Taken in context with other recent clinical and observational studies exploring treatment burden or routine follow‐up, our survey respondents' comfort with minimal lung function levels (average FEV_1_pp ≥ 67.03 ± 21.08%) are similar to PwCF and other researchers' perception on the minimally safe FEV_1_pp for study inclusions.

In terms of annual visits, all cohorts endorsed a slightly lower number of acceptable annual visits (range: 2.17–2.79 visits per year) compared to the previously recommended quarterly visits for routine care. Adult providers demonstrated more comfort with a slightly lower minimum number of annual visits when compared to PwCF and their pediatric counterparts. These differences may be due to pediatricians working with a healthier population of PwCF, thus striving to maintain the highest baseline status prior to their transition from the pediatric to adult clinic. Some PwCF also may find comfort in the traditional quarterly check‐in. Prior to HEMT, quarterly visits were the mainstay of CF care guidelines [11]. As PwCF have improved clinically with the use of HEMT, there has been a reduction in routine follow‐up in the population, with the average clinical encounters per year dropping from 4.76 in 2019 to 3.83 in 2024 (6)]. In response, the recent CFF position paper noted that reductions in clinic visit frequency may be appropriate with careful consideration on an individualized basis [12].

One of the major complications PwCF experience are PExs. PExs can be quite severe and are associated with a stepwise decline in lung function, with 25% of PwCF failing to recover to 90% of their baseline FEV_1_pp following an event [19]. Interestingly, based on survey responses, adult providers placed a stronger value on considering prior PExs requiring IV antibiotics than PwCF. Since the general use of HEMT, PExs in the CF population have significantly decreased. The annual average PEx events experienced by PwCF on HEMT dropped significantly from 0.8/year in 2019 to just 0.2/year in 2024 [6]. PwCF, pediatric, and adult provider survey participants felt comfortable in discussing altering visit frequency if they had a history of approximately 2 or fewer PExs requiring oral antibiotics in the previous year. The difference in considering PExs could be due to a number of reasons, including recall bias, optimism bias, or even just limited experience with PExs for some of the respondents.

Both PwCF and pediatric providers, compared to adult providers, did not differ in opinions on the importance of lung function, respiratory symptoms, co‐morbidities, HEMT, or medication adherence. However, a general trend was that PwCF, in comparison to adult and pediatric providers, were more willing to place less importance on the factors involved in determining the timing of the next clinic visit. This may reflect the importance PwCF place on the burden of clinic visits relative to their symptoms and state of health. Even well before the widespread availability of HEMT, Byczkowski et al. found that self‐perception of “good health” in a cohort of pediatric and adult PwCF was the most common reason associated with low importance ratings assigned to routine care visits [20]. Nevertheless, the profound impact HEMT has had on symptom burden, lung function, overall health, and advancing median survival is hard to minimize [6, 21]. This may be one of the reasons some PwCF downplayed some factors as less important when considering clinic visits.

PwCF, pediatric providers, and adult providers are in agreement with the importance that they placed on daily respiratory symptoms in discussing altering visit frequency. When one narrows the symptoms specifically to cough, PwCF and adult providers felt similarly based on the severity of cough. However, pediatric providers felt differently than adult providers on the degree of cough that would allow for one to safely extend the interval between clinic visits. This difference is likely due to pediatric providers' concern that increased coughing rates may be associated with increased disease severity or an acute PEx. This concept has been validated during both clinical stability and PExs [22, 23]. More recently, Zhang et al. demonstrated that following initiation of HEMT, patients had a reduction in the 24‐h cough counts [24]. Further, since widespread HEMT use, the degree of pulmonary and non‐pulmonary symptoms has decreased significantly [25, 26]. It seems prudent to consider symptom burden, but this domain is perhaps less prevalent than it has been in the past and potentially not weighted as heavily when compared to other metrics such as lung function stability and PExs.

PwCF experience many diagnoses that can potentially affect their health. Most of these involve infections or complications related to their lungs. CF providers were engaged to share their opinions on common CF diagnoses and the degree to which the presence of those could alter their tolerance for extending routine follow‐up. Pediatric practitioners expressed that prior history of *Burkholderia*, uncontrolled CFRD, TXP, and NTM colonization and/or infection were relevant contraindications, while adult providers did not feel as strongly. This difference may be related to perceived stability in this population despite these diagnoses. Pediatric practitioners were less likely to include pregnancy as an important factor when considering adjusting routine follow‐ups than their adult medicine colleagues. This difference may be due to the limited number of pregnancies in the pediatric population [27, 28].

Some of the limitations of the survey are its small sample size for both the limited number of healthcare providers and PwCF who completed it. While the survey was distributed through multiple CF community electronic communications, survey respondents were nonetheless a convenience sampling. Further, as no identifiable information was collected from survey participants, there is a potential for sample bias. No PwCF respondents endorsed actively attending a pediatric clinic, and therefore, it is unlikely any survey responses were from pediatric PwCF or PwCF parents/caregivers. This significantly limits the applications of the survey results for the pediatric population. Baseline demographics data were not collected from PwCF completing the survey, thus we do not know about their state of health, which may have affected their responses or subsequent interpretation. While the survey was developed through multiple iterations with input from PwCF, clinicians and survey experts, the survey is novel and not validated. It is plausible that there are other factors that were not addressed by this survey that still may be pertinent in discussing changes to visit frequency, including access to telehealth/video resources, tolerability to HEMT, objective monitoring, *etc*. Finally, it is important to note this survey was designed to gauge the importance PwCF and healthcare providers placed on clinical metrics when considering alterations to routine follow‐up. It was not designed to explore the actual safety and tolerability of routine care adjustments. For example, significantly reducing clinic visits without remote monitoring may increase the risk for delayed detection of *Pseudomonas* acquisition, which can affect eradication success [29]. The safety and tolerability of adjusting routine clinical follow‐up in PwCF across the age spectrum and disease severity is an important aspect of research that remains unanswered and must be further studied.

## 5 Conclusion

There is great interest in adapting the CF care model to fit with the current therapeutic environment within the CF community. Both PwCF as well as CF providers express some level of comfort when considering alterations to visit frequency. The major criteria that would be needed in this tool are the use of HEMT, meeting of a certain baseline level lung function, minimal to mild respiratory symptoms, a limited number of PExs that require IV and/or oral abx in the previous year, and exclusion of certain higher‐risk diagnoses. Future directions are to use this summative data to develop a pragmatic approach to having a shared discussion regarding routine care follow‐up. Importantly, future research is needed to explore the practicality and safety of adjusting routine clinical follow‐up across the CF community.

## Author Contributions

**William R. Hunt:** conceptualization, methodology, formal analysis, writing – original draft, writing – review and editing, funding acquisition, visualization, validation. **Christian Merlo:** conceptualization, methodology, writing – review and editing. **Ashley Keller:** investigation, writing – review and editing, resources, project administration. **Karen Lowe:** writing – review and editing, conceptualization. **Kristin Riekert:** writing – review and editing, methodology, conceptualization, investigation. **James D. Finklea:** methodology, conceptualization, data curation, investigation, software, formal analysis, funding acquisition, writing – original draft, writing – review and editing, project administration, visualization, validation, supervision, resources.

## Conflicts of Interest

The authors declare no conflicts of interest.

## Supporting information


Supporting File

## Data Availability

The data that support the findings of this study are available from the corresponding author upon reasonable request.
