# Supplementary material for: Meaningful Criteria to Persons Living With Cystic Fibrosis and Their Healthcare Providers in Helping Determine Adjustments to Routine Clinical Follow‐Up
Source: Pediatr Pulmonol. 2026 Jul 10;61(7):e71729. doi: 10.1002/ppul.71729 (PMC13352560; doi:10.1002/ppul.71729)
Supplement: Supplementary file 1 — Supporting File [file PPUL-61-0-s001.docx]

**CF CARE MODEL Visit Tool Medical Provider Survey**

After the approval of elexacaftor/tezacaftor/ivacaftor, people with cystic fibrosis (PwCF) had fewer CF clinic visits each year. Fortunately, the CF Foundation registry data showed that PwCF’s lung function did not change over time. The number of pulmonary exacerbations requiring intravenous antibiotics also was low. The CF community is wondering if PwCF can have fewer in-person visits without harming their health. There is no research data to show which PwCF can safely reduce in-person visits. Therefore, we are asking providers and PwCF for their opinions about what makes them feel comfortable in extending the time between routine CF clinic visits.

1. What type of population do you provide care for?
2. Pediatric
3. Adult
4. Both
5. For a person with CF who is clinically stable in your judgment, what is the minimum number of CF clinic visits per year that is adequate to maintain one’s health and lung function? ______
6. For an established person with CF who in your judgment is currently at their “baseline” and clinically stable, how important are the following to consider when deciding if it is safe to extend the clinic visit interval to more than 3 months?

Not at all A little Somewhat Greatly Completely

1. Lung function □ □ □ □ □
2. Pulmonary exacerbation history □ □ □ □ □
3. Respiratory symptoms □ □ □ □ □
4. Other co-morbidities □ □ □ □ □
5. Prescribed HEMT or not □ □ □ □ □
6. Adherence to Therapies □ □ □ □ □
7. Social determinants of health □ □ □ □ □
8. Other Criteria (list and rate) □ □ □ □ □

Deciding whether to extend the clinical visit interval involves thinking about many different health and personal factors cumulatively. However, please do your best to answer the following questions about each factor on its own. Please assume the person with CF is at their “baseline” and is clinically stable:

1. How many months do/would you want pwCF to be on HEMT (ivacaftor or elexacaftor/tezacaftor/ivacaftor) to feel safe to increase the time to the next clinic visit to more than 3 months? _______months
2. What is the lowest baseline FEV1 percentage predicted over the last 12 months for you to consider it safe to increase the time to the next clinic visit to more than 3 months?

__________ FEV1pp

1. What is the maximum number of pulmonary exacerbations requiring **IV** antibiotics pwCF could have in the last 12 months for you to consider it safe to increase the time to the next clinic visit to more than 3 months? _____ exacerbations
2. What is the maximum number of pulmonary exacerbations requiring **oral** antibiotics pwCF could have in the last 12 months for you to consider it safe to increase the time to the next clinic visit to more than 3 months? **________** exacerbations
3. What is the maximum level of “baseline” respiratory symptoms pwCF could have for you to consider it safe to increase the time to the next clinic visit to more than 3 months?
4. No pulmonary symptoms (e.g., never coughs or coughs once every few days)
5. Minimal pulmonary symptoms (e.g., coughs a few times during the day)
6. Mild pulmonary symptoms (e.g., coughs only in the morning after waking up)
7. Moderate pulmonary symptoms (e.g., coughs several times a day)
8. Persistent pulmonary symptoms (e.g., coughs at least once to multiple times every hour)
9. Which comorbidities would decrease the likelihood that you would consider it safe to increase the time to the next clinic visit to more than 3 months? (select all that apply)

**Infections**

1. Active Treatment for Allergic Bronchopulmonary Aspergillosis
2. Culture positive for *Burkholderia cepacia* complex
3. Culture positive for fungal (*Trichosporon* or *Scedosporium*) pathogens
4. Culture positive for Nontuberculous mycobacterium

**Pulmonary**

1. Uncontrolled Asthma
2. Chronic hypercapnic respiratory failure
3. Chronic hypoxic respiratory failure
4. Massive Hemoptysis in the last 12 months
5. Pneumothorax in the last 12 months

**Mental Health**

1. Moderate/severe Depression symptoms
2. Moderate/severe Anxiety symptoms

**Gastrointestinal**

1. Body Mass Index ≤ 18.5 kg/m^2^
2. Body Mass Index ≥ 30 kg/m^2^
3. Distal Intestinal Obstruction Syndrome in the last 12 months
4. Constipation

**Other**

1. Chronic sinusitis
2. Cystic Fibrosis related diabetes with an A1c ≥ 7.5
3. History of solid organ or hematological transplantation
4. Pregnancy
5. Other (open-ended responses)__________

**CF CARE MODEL Visit Tool People with CF Survey**

After the approval of elexacaftor/tezacaftor/ivacaftor, people with CF (pwCF) had fewer CF clinic visits each year.  Fortunately, the CF Foundation registry data showed that pwCF's lung function appeared stable. The number of
pulmonary exacerbations requiring intravenous antibiotics also was low. The CF community is wondering if pwCF could have fewer in-person visits without harming their health. There is no research data to show which pwCF can safely reduce in-person visits. Therefore, we are asking providers and pwCF for their opinions about what makes them more or less comfortable extending the time between routine CF clinic visits

1.What type of population do you provide care for?

- 1. Pediatric
  2. Adult
  3. Combined

2. Do you take a highly effective modulator therapy (HEMT (Kalydeco or Trikafta))?

A. Yes

B. No

3. If you are feeling well, what is the minimum number of CF clinic visits per year that you will need to attend to stay healthy and keep your lung function at the same level?

4. If you are feeling at your “baseline” and clinically stable, how important are the following to consider when deciding if it is safe to extend the clinic visit interval to more than 3 months?

Not at all A little Somewhat Greatly Completely

1. Lung function □ □ □ □ □
2. Pulmonary exacerbation history □ □ □ □ □
3. Respiratory symptoms □ □ □ □ □
4. Other co-morbidities □ □ □ □ □
5. Prescribed HEMT or not □ □ □ □ □
6. Adherence to Therapies □ □ □ □ □
7. Social determinants of health □ □ □ □ □
8. Other Criteria (list and rate) □ □ □ □ □

When deciding if you can increase the time to the next clinic visits you must think about many things that can cause a change in your health and daily lives. Please do your best to answer the following questions about each factor on its own. Please assume you are “feeling well” and you have your normal level of symptoms.

5. What is the lowest lung function (FEV1 percentage predicted) you could have and still feel it is safe to increase the time to the next clinic visit to more than 3 months? _____

6. What is the maximum number of courses of IV antibiotics you can have in the last 12 months and still feel it is safe to increase the time to the next clinic visit to more than 3 months? ____

7. What is the maximum number of pulmonary exacerbations requiring oral antibiotics you could have in the last 12 months and still feel it is safe to increase the time to the next clinic visit to more than 3 months? _____

8. What is the maximum level of "normal" respiratory symptoms you could have and still feel it is safe to increase the time to the next clinic visit to more than 3 months?

1. No pulmonary symptoms (e.g., never coughs or coughs once every few days)
2. Minimal pulmonary symptoms (e.g., coughs a few times during the day)
3. Mild pulmonary symptoms (e.g., coughs only in the morning after waking up)
4. Moderate pulmonary symptoms (e.g., coughs several times a day)
5. Persistent pulmonary symptoms (e.g., coughs at least once to multiple times every hour)
